# Multidrug Resistance Modulation Activity of Silybin Derivatives and Their Anti-Inflammatory Potential

**DOI:** 10.3390/antiox9050455

**Published:** 2020-05-25

**Authors:** Simona Dobiasová, Kateřina Řehořová, Denisa Kučerová, David Biedermann, Kristýna Káňová, Lucie Petrásková, Kamila Koucká, Radka Václavíková, Kateřina Valentová, Tomáš Ruml, Tomáš Macek, Vladimír Křen, Jitka Viktorová

**Affiliations:** 1 Department of Biochemistry and Microbiology, University of Chemistry and Technology Prague, Technická 5, CZ 166 28 Prague, Czech Republic; dobiasoo@vscht.cz (S.D.); rehorova@vscht.cz (K.Ř.); kuceroad@vscht.cz (D.K.); astriik@gmail.com (K.K.); rumlt@vscht.cz (T.R.); tomas.macek@vscht.cz (T.M.); 2 Laboratory of Biotransformation, Institute of Microbiology of the Czech Academy of Sciences, Vídeňská 1083, CZ 142 20 Prague, Czech Republic; david.biedermann@gmail.com (D.B.); petraskova@biomed.cas.cz (L.P.); kata.valentova@email.cz (K.V.); kren@biomed.cas.cz (V.K.); 3 Toxicogenomics Unit, National Institute of Public Health, Šrobárova 49, CZ 100 00 Prague, Czech Republic; kamila.koucka@lfp.cuni.cz (K.K.); radka.vaclavikova@szu.cz (R.V.); 4 Laboratory of Pharmacogenomics, Biomedical Center, Faculty of Medicine in Pilsen, Charles University, Alej Svobody 1655, CZ 323 00 Pilsen, Czech Republic

**Keywords:** silybin, dehydrosilybin, immunomodulation, P-glycoprotein, doxorubicin resistance, cytokines, expression profile, acetylcholinesterase inhibition

## Abstract

Silybin is considered to be the main biologically active component of silymarin. Its oxidized derivative 2,3-dehydrosilybin typically occurs in silymarin in small, but non-negligible amounts (up to 3%). Here, we investigated in detail complex biological activities of silybin and 2,3-dehydrosilybin optical isomers. Antioxidant activities of pure stereomers A and B of silybin and 2,3-dehydrosilybin, as well as their racemic mixtures, were investigated by using oxygen radical absorption capacity (ORAC) and cellular antioxidant activity (CAA) assay. All substances efficiently reduced nitric oxide production and cytokines (TNF-α, IL-6) release in a dose-dependent manner. Multidrug resistance (MDR) modulating potential was evaluated as inhibition of P-glycoprotein (P-gp) ATPase activity and regulation of ATP-binding cassette (ABC) protein expression. All the tested compounds showed strong dose-dependent inhibition of P-gp pump. Moreover, 2,3-dehydrosilybin A (30 µM) displayed the strongest sensitization of doxorubicin-resistant ovarian carcinoma. Despite these significant effects, silybin B was the only compound acting directly upon P-gp in vitro and also downregulating the expression of respective MDR genes. This compound altered the expression of P-glycoprotein (P-gp, *ABCB1*), multidrug resistance-associated protein 1 (MRP1, *ABCC1*) and breast cancer resistance protein (BCRP, *ABCG2*). 2,3-Dehydrosilybin AB exhibited the most effective inhibition of acetylcholinesterase activity. We can clearly postulate that silybin derivatives could serve well as modulators of a cancer drug-resistant phenotype.

## 1. Introduction

**Figure 1:**
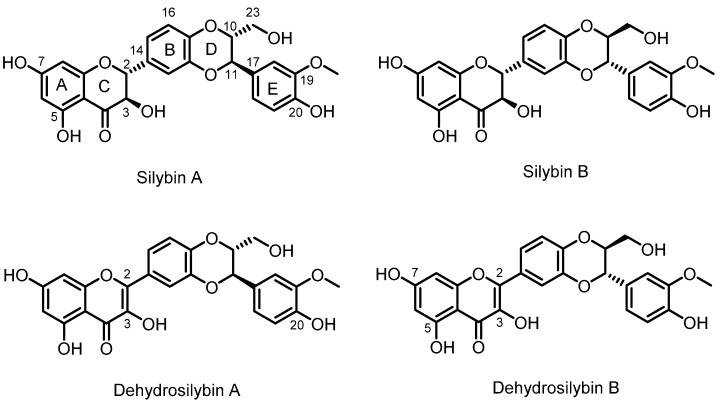
Structures of silybin diastereomers and 2,3-dehydrosilybin enantiomers, which occur in milk thistle.

Silybin (also denoted as silibinin) represents the major flavonolignan of silymarin complex isolated from *Silybum marianum* (L.) (milk thistle) cypselae [1]. Other flavonolignans, which form a substantial part of this polyphenolic fraction, are isosilybin, silydianin and silychristin. Natural silybin is a quasi-equimolar mixture of two diastereomers: silybin A and silybin B (Figure 1) [2,3,4]. For the preparatory separation of optically pure diastereomers, a lipase catalyzed method was developed combining acylation and alcoholysis [5]. Virtually all silymarin preparations contain non-negligible amounts of 2,3-dehydrosilybin (in the form of two enantiomers), which is formed by oxidation of silybin in the plant or during silymarin processing and storage [3]. Similarly, a base-catalyzed preparation of optically pure enantiomers, 2,3-dehydrosilybin A and B, from silybin and isosilybin, was described previously [6]. Despite their minor occurrence, 2,3-dehydroflavonolignans were shown to have more than one order of magnitude higher antioxidant [7] and anticancer [8] effects than their parent flavonolignans. The presence of 2,3-double-bond in 2,3-dehydrosilybin confers considerably higher antioxidant potency, resulting in 25-fold better radical scavenging and 10-fold better inhibition of lipid peroxidation compared to silybin [7,9]. Other benefits of 2,3-dehydrosilybin, especially cyto- and neuro-protection, have been described in several studies [1,4].

In recent studies, both silybin and 2,3-dehydrosilybin showed promising results not only in prevention of chronic liver damage [10], but also in anti-inflammatory and antifibrotic therapies [11]. In hepatic stellate cells (HSC), silybin decreased cell proliferation induced by platelet-derived growth factor (PDGF) and blocked de novo synthesis of collagen type I by reducing transforming growth factor-β1 (TGF-β) [12]. Furthermore, silybin and its congener silychristin inhibited IL-1β-induced formation of blood platelet-leukocyte aggregates in whole blood samples. Moreover, silybin reduced the release of pro-inflammatory cytokines IL-2, TNF-α, and INF-ƴ in a dose-dependent manner [11]. The cytotoxic and genotoxic effect of silybin was tested on blood platelets, peripheral blood mononuclear cells (PBMCs) and a human lung cancer cell line (A549). Even at concentrations up to 100 µM, silybin did not exhibit any toxic effect on these cell lines. Additionally, a protective effect of silybin was observed on cellular mitochondria with a reduction of spontaneous mitochondrial DNA (mtDNA) damage at 50 µM in the A549 cell line [13]. Moreover, 2,3-dehydrosilybin displayed greater ability than silybin to modulate skin inflammation by influencing the cytokines release in human dermal fibroblasts (HDF) [14]. On the other hand, from several flavonolignans tested, only 2,3-dehydrosilybin was identified as a potential phototoxic compound for HDF and human keratinocytes (HaCaT) [15].

Another important aspect of biological activity of silymarin flavonolignans is their anticancer potential. Nowadays, proposed cancer treatment regimens involve a new approach using natural compounds with limited or no cytotoxic effects on healthy cells, exploiting synergistic or adjuvant effects. Silybin is a typical example of such compound as it effectively inhibited overexpression of epidermal growth factor receptor (EGFR) and could be a potential target for therapeutic efficacy [16,17]. Recently, silymarin has been found to act against various types of malignant changes’ pathologies—skin, prostate, lung and ovarian cancer [18]. The combination of paclitaxel and 50 µM silybin in a human adenocarcinoma ovarian cancer cell line (SKOV-3) led to enhancement of therapeutic effectivity [19].

Due to excessive drug usage, healthy or malignant cells may change their sensitivity towards exogenous substances by an increased expression or higher activity of transport pumps from ATP-binding cassette (ABC) superfamily, especially P-glycoprotein (P-gp). This situation can be overcome by competitive, non-competitive or allosteric P-gp inhibition or via affecting P-gp expression [20]. Silybin seems to be a prospective compound in this regard, as pre-incubation of P-gp-positive small-cell lung cancer (VPA17) cells at 30 µM for 5 days led to decreased IC_50_ (concentration halving the viability) of etoposide [21]. Additionally, treatment of an ovarian carcinoma cell line (A2780/DPP) resistant to cisplatin and taxol with 50 µM silybin enhanced sensitivity of the cells to both drugs [22]. We have recently published modulation of P-gp expression by other flavonolignans, such as 2,3-dehydrosilychristin and anhydrosilychristin [23].

This study is aimed at a complex evaluation of biological activities of silybin and 2,3- dehydrosilybin pure stereomers, especially antioxidant, anti-inflammatory, inhibition of acetylcholinesterase activities and modulation of P-gp efflux pump, with emphasis on the comparison of the activities of individual, optically pure isomers.

## 2. Materials and Methods

### 2.1. Analytical Standards and Chemicals

2,2’-azo-*bis*-(2-Methylpropionamidine) dihydrochloride (AAPH, Sigma-Aldrich, St. Louis, MO, USA), 2´,7´-dichlorofluorescein diacetate (DCFH-DA, Sigma-Aldrich), 5,5′-dithio-*bis*-2-nitrobenzoic acid (DTNB, Sigma-Aldrich), 100 × antibiotic antimycotic solution (Sigma-Aldrich), acetylcholinesterase, acetylcholine chloride (Sigma-Aldrich), doxorubicin hydrochloride (sold under the trade name Adriamycin, Sigma-Aldrich), Dulbecco’s Modified Eagle’s medium—high glucose (DMEM, Sigma-Aldrich), Eagle’s minimum essential medium (EMEM, Sigma-Aldrich), Essential Medium Eagle no phenol red (MEM, Sigma-Aldrich), fetal bovine serum (FBS, Sigma-Aldrich), fluorescein (Sigma-Aldrich), Griess reagent modified (Sigma-Aldrich), indomethacin (Sigma-Aldrich), Interleukin-6 Mouse uncoated enzyme-linked immuno sorbent assay (ELISA) kit (Invitrogen, Thermo Fisher Scientific, Waltham, MA, USA), L-glutamine solution (Sigma-Aldrich), lipopolysaccharides from *Escherichia coli* O111:B4 (LPS, Sigma-Aldrich), Mouse TNF alpha uncoated ELISA kit (Invitrogen, Thermo Fisher Scientific, Waltham, MA, USA), quercetin (Sigma-Aldrich), resazurin sodium salt (Sigma-Aldrich), P-gp-Glo assay system (Promega, Madison, WI, USA), Trizol Reagent (Thermo Fisher Scientific, Waltham, MA, USA) and trypsin- ethylenediaminetetraacetic acid (EDTA) solution (Sigma-Aldrich) were used.

Silybin was isolated from silymarin (Liaoning Senrong Pharmaceuticals, Panjin, China, batch 120501) by suspension in methanol and filtration yielding solid silybin AB. The silybin diastereomers were separated as previously described [5] using the diastereomeric enzymatic resolution with immobilized lipase B from *Candida antarctica* (Novozyme 435, Novo-NORDISK, Copenhagen, Denmark). 2,3-Dehydrosilybin (racemate or enantiomers) was prepared as described previously [5]. Briefly, silybin was dissolved in glacial acetic acid and anhydrous potassium acetate and iodine was added. After refluxing for 3 h, the mixture was diluted with water and filtered. The product (solid) was refluxed in a mixture of ethanol/hydrochloric acid to deacetylate partially acetylated product.

### 2.2. Antioxidant Capacity

Antioxidant capacity of samples was determined by ORAC (oxygen radical absorption capacity) and CAA (cellular antioxidant activity) assays. ORAC assay was realized by the previously described protocol [24]. Briefly, 50 µL of the fluorescein solution (1.6 µg/mL in phosphate-buffered saline (PBS), pH 7.4) was added by a dispenser (MultiFlo Microplate Dispenser, BioTek Instrument, Winooski, VT, USA) into each well of a 96-well plate. Subsequently, 2 µL of tested compound in the concentration range 1.25–20 µM and 23 µL of PBS were added to the wells. The plate was incubated for 15 min at 37 °C and then 25 µL of AAPH (60 mg/mL) was added to each well. AAPH was replaced with PBS for the negative control. Immediately, the fluorescence was recorded by the microplate reader (SpectraMax i3 Multi-Mode Detection Platform, Molecular Devices, San Jose, CA, USA) for 2 h in 5 min steps (excitation/emission 485/535 nm).

For the CAA assay, human hepatocellular carcinoma HepG2 cells (ATCC, Manassas, VA, USA) were seeded into a 96-well microplate at a density of 1 × 10^5^ cells/well in EMEM medium supplemented with 10% of FBS, 2 mM L-glutamine and 1 × antibiotic antimycotic solution. The cells were maintained in a CO_2_ incubator (5% CO_2_, 37 °C, Memmert GmbH & Co, Büchenbach, Germany). The following day, the plates were washed with PBS and new fresh DMEM was added into each well supplemented with tested samples in the concentration range of 6.25–100 µM and fluorescent probe DCFH-DA (12.5 mg/L). After 1 h of incubation at 37 °C, the medium was replaced with AAPH solution (0.16 mg/mL in PBS), except for the negative control, where PBS was added instead. Directly, the fluorescence was recorded for 2 h in 5 min steps at 485/540 nm excitation/emission (ex./em.).

### 2.3. Immunomodulatory Activity

Immunomodulatory effect of tested compounds was determined as their ability to decrease nitric oxide (NO) production by LPS-stimulated macrophages (RAW 264.7, Sigma-Aldrich). Briefly, the cells were seeded into the 96-well plate at a density of 1 × 10^6^ cells/mL in DMEM medium (supplemented with 2 mM L-glutamine and 1 × antibiotic antimycotic solution). After 48 h, the cells were washed with PBS. The MEM medium enriched with LPS (1 µg/mL) was added to the wells, together with the samples in the concentration range of 6.25–100 µM at a final volume of 100 µL. Indomethacin, a non-steroidal anti-inflammatory drug, was tested as a positive control in the concentration range 6.25–100 µM. After 24 h, the medium was used for the quantification of NO and inflammatory cytokines.

To determine the NO production, the medium was mixed with Griess reagent (0.04 g/mL, prepared in deionized water) in a 1:1 ratio. After 15 min, absorbance was recorded at 540 nm. To determine cell viability, the resazurin solution (0.03 mg/mL in PBS) was added to the cells. After 2 h of incubation, fluorescence was recorded (560/590 nm, ex./em.).

To determine the level of inflammatory cytokine TNF-α, macrophages were pre-cultivated with the tested compounds (12.5–50 µM) for 24 h. Thereafter, the plates were washed with PBS and new MEM medium enriched with LPS (1 µg/mL) was added to the wells. After 2 h of incubation, the plates were centrifuged (1000× *g*; 10 min, 4 °C) and then the medium was removed and frozen to −80 °C. Prior to the detection, the samples were diluted in ELISA diluent in a 1:10 ratio.

To determine the level of inflammatory cytokine IL-6, macrophages were cultivated with LPS (1 µg/mL) and the tested compounds (7–50 µM) for 6 h. After incubation, the plates were centrifuged (1000× *g*; 10 min, 4 °C) and then the medium was removed and frozen to −80 °C. Prior to the detection, the samples were diluted in ELISA diluent in a 1:10 ratio. Production of the cytokine was determined by uncoated ELISA performed according to the manufacturer’s instructions.

### 2.4. Modulation of P-Glycoprotein and Acetylcholinesterase Activity

The procedure and the dilution of the reagents were performed according to the manufacturer’s instructions. Briefly, the human recombinant P-gp-containing membrane (25 µg) was mixed in a 96-well plate with P-gp–Glo™ Assay buffer (control), verapamil (P-gp substrate, positive control, 0.2 mM), Na_3_VO_4_ (P-gp inhibitor, negative control, 0.1 mM), ATP standards (to construct the calibration curve, 0.375–3 mM) or samples (2.5 µL, in the concentration range 0.7–20 mM). After that, MgATP (5 mM) was added to total volume of 50 µL to each well to initiate the ATPase reaction. The plate was incubated for 1 h at 37 °C. Thereafter, the reaction was stopped by adding 50 µL of ATP detection reagent and incubated at room temperature for 20 min. The remaining non-metabolized ATP was detected as a luciferase-generated signal.

Using linear regression, the standard ATP curve was designed, and by subsequent interpolation of relative luminescence (RLU) of standards, the amount of ATP consumed in the samples was recalculated according to the manufacturer’s guidelines [25].

The activity of acetylcholinesterase was measured by the Ellman colorimetric method [26]. A stock solution of acetylcholinesterase was prepared in PBS (0.057 U/mL), 100× diluted and stored in a freezer (−20 °C). Further, the solution of DTNB (5,5′-dithio-*bis*-2-nitrobenzoic acid, 50 µM) and ATC (acetylcholine chloride, 50 µM) was prepared. PBS (45 µL), the solution of acetylcholinesterase (10 µL) and the compounds (0.2–200 µM) were added to the wells of a 96-well plate and incubated for 15 min at 37 °C. Afterwards, 10 µL of DNTB and ATC solution was added, and the reaction was launched. After the addition of reagents, the absorbance at 412 nm was recorded for 10 min every minute.

### 2.5. Sensitization of the MDR Cell Line

A multidrug resistant ovarian sub-line resistant to doxorubicin (A2780/DOX, Sigma-Aldrich) was cultivated in DMEM medium with 10% FBS and 1 × antibiotic antimycotic solution. Every other passage, doxorubicin at the final concentration of 0.1 µM was added to the culture medium to maintain the resistance of the cell line. To perform experiments, the cells were seeded at 1 × 10^5^ cell/mL into the wells of a 96-well plate and incubated for 24 h. Afterwards, the cells were washed with PBS and fresh DMEM medium with tested compounds at 10, 20 and 30 µM were added to each well, except for the control wells. Subsequently, the cells were exposed to increasing concentrations of doxorubicin (0.6–20 µM) for 72 h. After incubation, the resazurin assay was performed as described above. The IC_50_ of doxorubicin was calculated for both the control and the samples and the fold change was counted up. If the resulting fold change was higher than 1, the treatment of the chemotherapeutic (doxorubicin) and tested compound indicate a mutual synergistic effect. The fold change lower than 1 means an antagonistic effect.

(1) $$\mathrm{FOLD}\;\mathrm{CHANGE}=\frac{{\mathrm{IC}}_{50}\;\mathrm{value}\;\mathrm{of}\;\mathrm{DOX}\;\mathrm{cells}\;\mathrm{treated}\;\mathrm{with}\;\mathrm{chemotherapeutic}\;}{{\mathrm{IC}}_{50}\;\mathrm{value}\;\mathrm{of}\;\mathrm{DOX}\;\mathrm{cells}\;\mathrm{treated}\;\mathrm{with}\;\mathrm{chemotherapeutic}\;\mathrm{and}\;\mathrm{compound}}$$

### 2.6. Effect of (2,3-dehydro)silybins on the Expression of MDR Phenotype-Associated Transporters

The A2780/DOX cell line was seeded into 5 cm Petri dishes with DMEM medium at 1 × 10^5^ cell/mL. After 24 h, the dishes were washed with PBS and new DMEM medium supplemented with doxorubicin (at concentration equal to IC_25_) and samples (10 µM) were added. After 48 h of treatment, the cells were washed with PBS and harvested using the standard trypsin-EDTA procedure. The harvested cells were centrifuged (3200× *g*; 10 min, 4 °C). The supernatant was discarded, and the pellets were washed with 1.5 mL of cooled PBS (5400× *g*; 10 min, 4 °C). The washing step was repeated with 1 mL of PBS and the cells were centrifuged again (10,000× *g*; 3 min, 4 °C). Afterwards, the pellets were resuspended in 1 mL of Trizol (Invitrogen, Carlsbad, CA, USA) and transferred into pre-cooled RNase-free microfuge tubes. The samples were stored at −80 °C.

A Quant-iT RiboGreen RNA Assay Kit (Invitrogen) was used to determine the RNA concentration using the Infinite M200 plate reader (Tecan, Männedor, Switzerland). The cDNA was prepared from 0.5 µg of total RNA using a RevertAid First Strand Synthesis cDNA Kit (MBI Fermentas, Vilnius, Lithuania). The amplification of the ubiquitin C gene fragment was used to determine the quality of cDNA.

The experiment was performed using the ViiA7 Real-Time polymerase chain reaction (PCR) System (Life Technologies, Camarillo, CA, USA) with a 384-well block. Before quantitative real-time PCR, the master mix was prepared by mixing 2 µL of 8 × diluted cDNA, 1.75 µL of RNase free water, 1 µL of 5 × HotFIREPol Probe quantitative PCR Mix Plus (Solis Biodyne, Tartu, Estonia) and 0.25 µL of specific 20 × TaqMan Gene Expression Assay (Life Technologies), giving 5 µL of final reaction volume. Cycling parameters were: initial hold at 50 °C for 2 min and denaturation at 95 °C for 10 min, followed by 45 cycles consisting of denaturation at 95 °C for 15 s and annealing/extension at 60 °C for 1 min. After each extension phase, the fluorescence values were acquired. Duplicate samples were used for measurement and samples with a standard deviation of duplicates > 1.0 cycle threshold (Ct) were re-analyzed. The real time PCR procedure was maintained according to MIQE (Minimum information for publication of quantitative real-time PCR experiments) guidelines [27]. The software REST 2009 (Qiagen, Hilden, Germany) served to compare relative transcript levels of genes in the cell line.

### 2.7. Data Processing and Statistical Analysis

The experiments were carried out in a respective number (n) of repetitions. The relative activity (RA) of samples within the individual assays was determined as:

(2) $$\mathrm{RA}\;\left(\%\right)=100*\frac{\mathrm{slope}\;\mathrm{of}\;\mathrm{sample}-\mathrm{average}\;\mathrm{slope}\;\mathrm{of}\;\mathrm{NC}}{\mathrm{average}\;\mathrm{slope}\;\mathrm{of}\;\mathrm{PC}-\mathrm{average}\;\mathrm{slope}\;\mathrm{of}\;\mathrm{NC}}$$

where PC represents positive control and NC represents negative control. The GraphPad Prism 7 software (GraphPad Software, San Diego, CA, USA) was used to calculate IC_50_ values using non-linear regression:

(3) $$Y=\frac{\mathrm{Bottom}+\left(\mathrm{Top}-\mathrm{Bottom}\right)}{1+10^{ˆ}\left(\left(\mathrm{LogIC}-X\right)*\mathrm{HillSlope}\right)}$$

The data were averaged and are presented with the standard error of the mean (SEM). The Excel *t*-test function was used to check statistical significance. The data were analyzed with one-way analysis of variance (ANOVA, Statistica 13, Tibco Software Inc., Tulsa, OK, USA) and Duncan’s post hoc test, where the differences between the groups were considered as statistically significant when *p* < 0.05.

## 3. Results

### 3.1. Antioxidant Capacity

**Figure 2:**
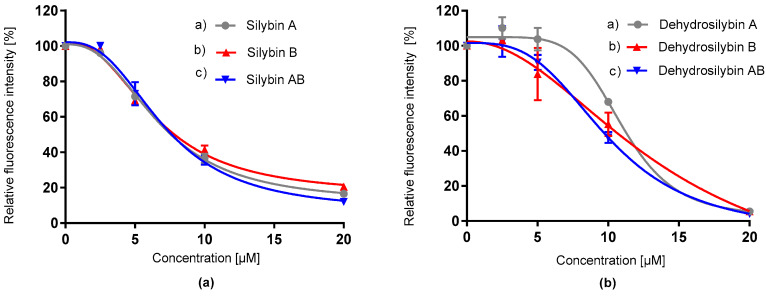
Relative oxygen radical absorption capacity (ORAC) of silybin diastereomers (**a**) and dehydrosilybin enantiomers (**b**). Data represent the average of three repetitions with corresponding standard error of the mean (SEM).

**Figure 3:**
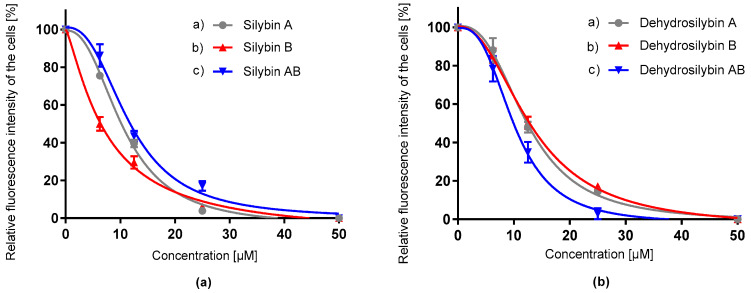
Relative oxygen radical absorption capacity of silybin diastereomers (**a**) and dehydrosilybin enantiomers (**b**) in cellular antioxidant activity assay (CAA, human hepatocellular carcinoma HepG2 cell line). Data represent the average of three repetitions with corresponding standard error of the mean.

The antioxidant potential of the tested compounds was recorded by two independent chemical methods. ORAC assay is a method that detects the total ability of the compounds to scavenge peroxyl radicals. In contrast, CAA, as a cell-based assay, quantifies the antioxidant potential of substances using the HepG2 cell line. All silybin and 2,3-dehydrosilybin isomers proved good antioxidant capacity in a dose-dependent manner in both assays (Figure 2, Figure 3, Table 1). Results from the ORAC assay showed that silybin and its diastereomers, as well as dehydrosilybin (enantiomers or racemate) (Figure 2), have almost the same IC_50_ values (Table 1). The antioxidant potential of the flavonolignans was compared with that of quercetin, which is known as a strong antioxidant. The *p*-value of the t-test exhibited differences between quercetin and other tested compounds in the CAA assay, but in ORAC, a significant difference was observed only between quercetin and dehydrosilybin samples. The *p*-value of the test revealed a difference between the group of silybin and dehydrosilybin isomers. In the CCA assay (Figure 3), 2,3-dehydrosilybin AB, silybin AB and its isomers achieved similar antioxidant capacity levels with little differences between IC_50_ values. The *p*-value differences between dehydrosilybin AB and enantiomers A and B were 0.008 and 0.004, respectively. In addition, in both assays, silybins seemed to provide better antioxidant activity compared to dehydrosilybins.

### 3.2. Anti-inflammatory Properties

**Figure 4:**
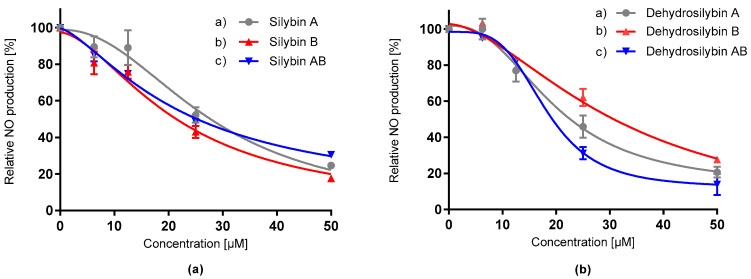
Effect of silybin diastereomers (**a**) and dehydrosilybin enantiomers (**b**) on relative production of nitrite oxide by lipopolysaccharide (LPS)-stimulated RAW 265.4 macrophages. Data represent the average of three repetitions with corresponding standard error of the mean.

The immunomodulation potential of the tested compounds was investigated on macrophage cells (RAW 264.7) induced by bacterial lipopolysaccharide (LPS). Anti-inflammatory effect was determined as the ability to decrease the production of the first-response molecule - NO (Figure 4). We demonstrated the ability of silybins and dehydrosilybins to inhibit the production of NO in a dose-dependent manner. Dehydrosilybin AB was the strongest modulator of inflammatory response (Table 2). The immunomodulation effect of dehydrosilybin B was approximately 40% lower compared to its diastereomeric mixture (*p* = 0.03). Indomethacin, a non-steroidal anti-inflammatory drug, as the positive control, provided the least significant immunomodulatory potential among the tested compounds.

**Figure 5:**
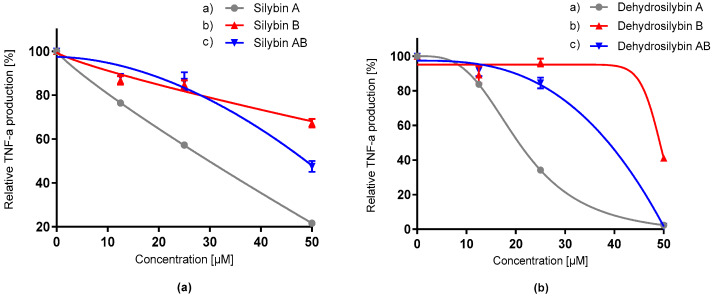
Effect of silybin diastereomers (**a**) and dehydrosilybin enantiomers (**b**) on relative production of TNF-α by LPS-stimulated RAW 265.4 macrophages. Data represent the average of four repetitions with corresponding standard error of the mean.

The potency of the tested flavonolignans to modulate the immune response was determined on LPS-induced macrophages by monitoring cytokine (TNF-α and IL-6) release as the first reaction, by which a healthy cell responds to the bacterial infection. For this experiment, the uncoated sandwich ELISA kit was used. ELISA provided high sensitivity in the detection of potential cytokine modulation. TNF-α, which is considered as the first signaling protein in the cytokine inflammation pathway, was investigated. The effect of pre-incubation by flavonolignans on the release of this pro-inflammatory cytokine was observed. All silybins and dehydrosilybins showed a dose-dependent protective effect that led to the decrease of TNF-α production (Figure 5, Table 3). Dehydrosilybin A and dehydrosilybin AB provided the most promising effect, followed by silybin A. The pre-treatment effect of various concentrations of flavonolignans is also represented as the level of released TNF-α (Table 4).

**Figure 6:**
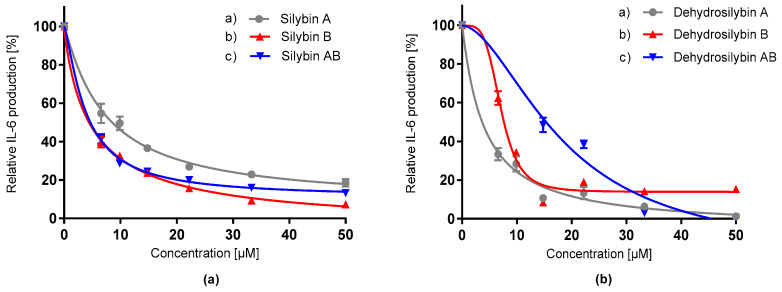
Effect of silybin diastereomers (**a**) and dehydrosilybin enantiomers (**b**) on relative production of IL-6 by LPS-stimulated RAW 265.4 macrophages. Data represent the average of four repetitions with corresponding standard error of the mean.

In the next step, the potential of silybins and dehydrosilybins to modulate the IL-6 cytokine production was determined on LPS-induced macrophages by the uncoated sandwich ELISA kit. All silybin and dehydrosilybin isomers suppressed IL-6 release in a dose-dependent manner (Figure 6, Table 5). The highest inhibition effect on IL-6 release was observed by dehydrosilybin A, followed by silybin AB and silybin B. The IL-6 modulation effect of dehydrosilybin A was approximately 70% higher in comparison with its diastereomeric mixture. The *p*-value of the *t*-test confirmed that there is no difference between silybin AB and silybin B, and also dehydrosilybin A. The treatment effect of various concentrations of flavonolignans is also represented as the level of released IL-6 (Table 5).

The regulation of pro-inflammatory cytokines by the flavonolignans was detected by ELISA. Using mouse TNF-α and IL-6 standards, the standard curves were constructed, and by subsequent interpolation of absorbance of standards, the amount of cytokines release (pg/mL) in the samples was recalculated with respect to the dilution factor. After treatment and LPS challenging of macrophages, a statistically significant increase of TNF-α and IL-6 was observed (Table 4 and Table 5). All the compounds tested showed the highest effect at 50 µM. At this concentration, dehydrosilybin A and dehydrosilybin AB decreased the release of TNF-α and IL-6 under the detection limit of the ELISA assay. With the decreasing concentration of the tested compounds, the most promising results were observed with dehydrosilybin A in both of the cytokine assays. 25 µM dehydrosilybin A decreased release of TNF-α almost 4 times (from 2954 pg/mL to 777 pg/mL). Additionally, all the compounds, except dehydrosilybin AB at the lowest concentration (7 µM), were able to inhibit the release of IL-6 at least 2 times (Table 5).

### 3.3. Modulation of P-gp and Acetylcholinesterase Activity

The P-gp–Glo Assay System is a bioluminescent ATPase activity assay. Compounds interacting with P-gp may increase or decrease its activity, resulting in a change of ATP consumption. The remaining non-metabolized ATP is detected as a luciferin-generated signal. Recombinant P-gp containing membrane fraction shows basal ATPase activity. Sodium orthovanadate (Na_3_VO_4_) as a negative control inhibits the consumption of ATP, resulting in a higher luminescent signal. On the other side, verapamil, as a substrate of P-gp, stimulates the activity of the efflux pump, which decreases the level of ATP. To determine the P-gp inhibition activity of substances, verapamil was added to the corresponding wells. In the presence of the tested compounds, verapamil is acting as a competitive substrate interfering with their transport. When luminescence of the sample is higher than the basal luminescence, the tested compound is considered as an activator. In contrast, a lower luminescence signal indicates a P-gp inhibitor.

**Figure 7:**
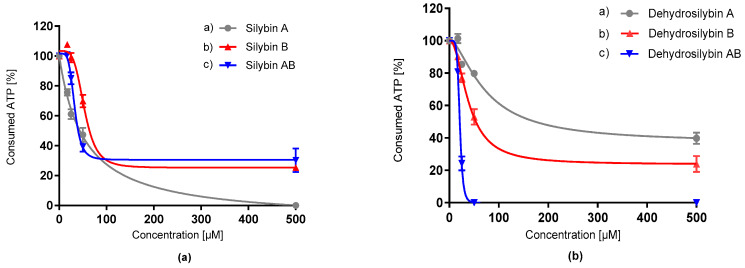
Modulation of P-glycoprotein (P-gp) ATPase activity by silybin diastereomers (**a**) and dehydrosilybin enantiomers (**b**). The assay was realized using the commercial P-gp-Glo assay system (Promega, Madison, WI, USA) including a membrane fraction with recombinant human P-gp. Data represent the average of three repetitions with corresponding standard error of the mean.

Specific P-gp activity was determined by using a standard ATP curve and calculated amount of consumed ATP (pmol) per minute. Silybin and dehydrosilybin isomers were tested for their capacity to inhibit verapamil-stimulated P-gp ATPase activity at a range of concentrations (Figure 7). Each of the tested compounds caused a dose-dependent decrease of ATP consumption. Silybin AB (Figure 7, Table 6) as a mixture of diastereomers A and B showed lower IC_50_, suggesting more potent inhibition of this efflux pump compared to its pure isomers. However, its oxidation product, dehydrosilybin AB, caused complete inhibition of ATP consumption at a 50 µM concentration. Therefore, dehydrosilybin AB could be an inhibitor binding directly to the P-gp ATP binding site or to the allosteric residue, affecting P-gp function. The *p*-value of the *t-*test exhibited no statistically significant difference between silybin AB and dehydrosilybin AB. However, the difference between dehydrosilybin AB and its enantiomers dehydrosilybin A and dehydrosilybin B was significant, with *p*-values 0.00008 and 0.008, respectively.

The acetylcholinesterase inhibitory activity of the compounds was determined by the Ellman method. The acetylcholine hydrolysis was detected by monitoring yellow 5-thio-2-nitrobenzoate anion, which was the result of the reaction with DTNB. Eserin, as the reversible inhibitor of acetylcholinesterase, was used in the reaction to compare the inhibition capacity of silybin and dehydrosilybin compounds. Positive control contained the enzyme with DTNB and the substrate. Negative control contained all the reagents except acetylcholine. The tested compounds inhibited the activity of acetylcholinesterase in a dose-dependent manner. The highest inhibition potential was observed with dehydrosilybins. The *t*-test indicated a statistically significant difference between racemic dehydrosilybin and its pure enantiomers, and also between silybin AB and its diastereomers A and B. In addition, the racemic mixture of dehydrosilybin inhibited the acetylcholinesterase activity 2.5× more than single isomers.

### 3.4. Sensitization of the Multidrug Resistant Cell Line

The ability of silybin and dehydrosilybin stereomers to inhibit P-gp ATPase activity was described in the previous section. Based on this fact, the P-gp-positive human adenocarcinoma cell line resistant to doxorubicin (A2780/DOX) was used to demonstrate the effect of compounds under in vitro conditions. First, A2780/DOX cells were incubated with silybins and dehydrosilybins to investigate the possibility of cytotoxic response (Table 7). Both dehydrosilybin stereomers demonstrated a stronger cytotoxic effect, resulting in a lower concentration required to lower the cell viability (IC_50_). Non-statistically significant differences between the racemic mixture and pure isomers B were found for both silybin and dehydrosilybin. However, isomers A were less active than isomers B for both dehydrosilybin and silybin.

To modulate the P-gp-positive A2780/DOX cell line, doxorubicin (concentration range 0.1–20 µM) and silybins or dehydrosilybins were added to culture medium. The concentration of applied compounds had been modified depending on previous results of cytotoxicity and P-gp inhibition assays. The addition of silybins and dehydrosilybins sensitized the resistant ovarian cells to doxorubicin: its IC_50_ was decreasing with increasing concentration of the flavonolignans (Table 8). At the lowest tested concentration (10 µM), dehydrosilybin AB (racemate) displayed the most significant effect. Also, the highest concentration of silybins (50 µM) made resistant cells more sensitive to doxorubicin. A significant difference was found between silybin AB and silybin A (*p* ≤ 0.002) and silybin AB and diastereomer B (*p* ≤ 0.001). The concentrations of dehydrosilybin A (50 µM), dehydrosilybin B (20 µM) and dehydrosilybin AB (20 µM) decreased the cell viability below 80%. Due to their direct cytotoxicity, these concentrations were excluded from the sensitization assay.

The sensitization rate was also evaluated (Table 9). When the resulting fold change was higher than 1, the treatment of the cells with doxorubicin and the tested compound indicated a mutual synergic effect. The fold change lower than 1 indicated an antagonistic effect. The results proved that all the compounds showed a dose-dependent synergistic effect with doxorubicin, resulting in the decrease of its IC_50_. The highest fold was observed with 30 µM dehydrosilybin A, followed by 50 µM silybin AB. These concentrations decreased the IC_50_ of doxorubicin almost 10×, which resulted in a change of the resistant phenotype of cancer cells to sensitive.

### 3.5. Inhibition of ABC Transporters Expression

Further, we were interested whether silybins and dehydrosilybins modulate the capacity of efflux transport pumps by regulation of their mRNA expression level. The MDR cell line (A2780/DOX) was analyzed for the expression profile of ABC superfamily genes. To compare the effect of treatment by tested compounds, the cells were incubated with 10 µM silybins and dehydrosilybins with or without addition of doxorubicin (IC_25_ = 0.94 µM). After 48 h of treatment, the most significant results were noticed on the resistant cells incubated in the medium only with the mentioned compounds (Appendix A). The expression profile of mRNA of almost the whole ABC superfamily was downregulated by silybin B. Only this isomer effectively downregulated the expression of all ABC genes related to the MDR phenomenon—ABCB1 (P-gp) by 79%, ABCC1 (MRP1) by 59%, ABCC2 (MRP2) by 66% and ABCG2 (BCRP) by 67%. In total, silybin B altered the expression of 24 ABC genes out of 42 tested. Dehydrosilybin enantiomers A and B slightly downregulated expression of ABCA2 by 22% (dehydrosilybin A), 11% (dehydrosilybin B) and also ABCG4 by 5% (enantiomer A) and 26% (enantiomer B), respectively. The diastereomeric mixture silybin AB increased the expression of 20 ABC genes. Interesting results were obtained when the cells were treated with the combination of the tested compounds and doxorubicin at IC_25_. The effect of silybin B was decreased due to the addition of doxorubicin, which seems to be a strong effector. In this case, silybin downregulated only four ABC genes and expression of six genes was increased. When silybin B and doxorubicin were added together, ABCB1, ABCB2 and ABCG2 genes were no more downregulated. Additionally, all the compounds caused overexpression of ABCA1, ABCA5, ABCB2, ABCC10 and ABCG4. In contrast, downregulation of the ABCD2 gene by all silybins and dehydrosilybins was observed. In our study, we focused on the changes in genes connected with the MDR phenotype of the ovarian carcinoma cell line, specifically on over-expression of the ABCB1 gene. From this perspective, silybin B was the only compound affecting both P-gp and other MDR pumps’ expression.

## 4. Discussion

Silymarin complex has been known for millennia for its beneficial biological effects on human health [28]. Silymarin components possess a wide range of biological properties, such as antioxidant, anticancer and anti-inflammatory activities [6]. In our study, the antioxidant capacity of silybin and 2,3-dehydrosilybin pure stereomers was determined as their ability to scavenge oxygen radicals to protect fluorescein from oxidation (ORAC) and also to scavenge radicals generated in the living cells (CAA). Isomers A and B of both silybin and dehydrosilybin were approximately equally active. In both assays, silybins and dehydrosilybins reduced the intracellular reactive oxygen species (ROS) level. In contrast to published data [8], no higher activity of dehydrosilybin compounds was observed. This fact may be associated with higher cytotoxicity of dehydroflavonolignans [29] or with the different methodological approach to antioxidant capacity determination [8].

Both applied assays represented a useful tool for screening of antioxidant scavenging of compounds. Despite this fact, neither of these two methods were able to predict the in vivo potential of tested compounds [30]. Moreover, many natural compounds, such as flavonolignans, usually act as antioxidants in vitro, but in fact, they activate various enzymatic systems in vivo—often at an expression level. The nutrient antioxidant potential is associated with activation of antioxidant and non-antioxidant enzymes, and especially activation of transcription factors such as Nrf2 (nuclear factor erythroid 2-related factor 2) and NF-ĸB (nuclear factor kappa-light-chain-enhancer of activated B cells) [28,30]. The essential role of the Nrf2 signaling pathway is to maintain the protection of oxidoreductases and their nucleophilic substrates, also called “nucleophilic tone’’. By the mechanism of “para-hormesis’’, nontoxic concentrations of compounds are able to active the transcription of antioxidant enzymes, resulting in protection from damage [31]. On the other side, several studies have shown the correlation between cancer progression and activation of the endogenous Nrf2 system by antioxidants [32]. An in vivo study on mouse KRAS (kirsten rat sarcoma viral oncogene homolog) has driven a lung cancer-demonstrated negative impact of long-term supplementation of *n*-acetylcysteine and vitamin E on metastasis formation. Antioxidants stabilized transcription factor BACH1 by inhibition of oxidative stress (Nrf2 pathway). BACH1, as a crucial player of glucose uptake regulation, has ultimately stimulated glycolysis-dependent growth of mouse metastasis [33]. Nrf2 factor participates as the important tumor suppressor against cell oncogenesis, but also may support cancer progression and drug resistance, depending on type, location and stage of carcinoma [34]. Therefore, we focused on more complex and biologically relevant assays as well.

Silymarin is, among others, considered as a strong anti-inflammatory agent, which inhibits activation of NF-ĸB in hepatocellular carcinoma (HepG2) [35]. NF-ĸB is a protein complex that controls transcription of DNA, cytokine production and cell survival. NF-ĸB has been known as one of the most important pro-inflammatory gene expression regulators, which also activates synthesis of cytokines such as TNF-α, IL-1β or IL-8. All of these cytokines were significantly suppressed by silybin in LPS-induced acute lung injury [36]. In addition, flavonoids demonstrated the ability to modulate inflammatory response by regulating the expression of pro-inflammatory cytokines (IL-6, TNF-α, IL-1β and COX-2) [37]. Some studies investigated the potential of silybin, dehydrosilybin and silychristin to regulate the release of inflammatory molecules [12,15,35,37]. Flavonoids, such as quercetin, had shown in vitro inhibition of NF-ĸB activation and subsequent inhibition of TNF-α release at a µM concentration range [38]. On the other side, TNF-α can be considered as a pluripotent cytokine, which can trigger the production of IL-6 [39] and also regulate NO expression, resulting in changes of NO production [40]. With respect to this situation, we demonstrated the ability of stereomeric mixtures and their pure stereomers to inhibit the production of inflammatory markers (NO) in a dose-dependent manner. Inhibition of TNF-α and IL-6 release of LPS-induced macrophages by tested compounds confirmed that they are effective immunomodulators of acute phase of cytokine release (TNF-α, IL-6). In line with this, the great potential of dehydrosilybin in LPS-induced primary human fibroblasts (HDF) was observed in a previous study as well. In a dose-dependent manner, dehydrosilybin inhibited secretion of IL-6 and IL-8 [14].

Application of natural and nontoxic products is highly desirable also in cancer treatment. During the therapy, the carcinoma cells may develop multi-drug resistance (MDR) in response to a specific drug or drug combination. However, there are many factors which contribute to the development of the MDR phenotype, such as increased activity of detoxification enzymes, changes in apoptosis genes of the Bcl-2 family and overexpression of several efflux pumps from the ABC family, especially P- glycoprotein [41,42]. In a few studies, the P-gp reversing activity of several flavonoids was evaluated [43,44,45] and the possibility of MDR modulation by flavonolignans and flavonoids was recently summarized [46]. Silymarin as a complex is generally known for its inhibitory effects on P-gp transporter [46,47,48]. Also, the combination of one silymarin component—quercetin with doxorubicin—significantly increased the doxorubicin sensitivity of doxorubicin-resistant breast cancer (MCF-7) cells and quercetin also decreased P-gp expression [49]. We noticed strong P-gp ATPase inhibition activity of all silybins and dehydrosilybins in a dose-dependent manner. The most effective outcome from P-gp inhibition was achieved with diastereomeric mixture of dehydrosilybin AB, that resulted in sensitization of a P-gp-positive ovarian carcinoma cell line resistant to doxorubicin. The observed higher activity of dehydrosilybin compared to silybin could be caused by the presence of the double bond on C-2,3 in ring B, which increases lipophilicity of dehydrosilybin, thus facilitating its interaction with the membrane proteins/lipids and enhancing the cytotoxic potential [50]. In agreement with our results, treatment of the ovarian carcinoma cell line (A2780/DPP) resistant to cisplatin and taxol with silybin also enhanced the sensitivity of the cells [22].

As described in our previous study [23], the resistance of the A2780/DOX cell line is connected to the over-expression of several ABC genes, especially ABCB1 (P-gp) and ABCC1 (MRP1). Therefore, the modulation of P-gp could be caused by inhibition of ATPase activity or by regulation of its expression. Our results showed that all silybin and dehydrosilybin isomers exhibit the ability to modulate P-gp activity by acting as competitive inhibitors. Additionally, silybin B also modulated the resistant phenotype by P-gp gene downregulation. Moreover, silybin B and enantiomers of dehydrosilybin downregulated expression of the ABCA2 gene. Interestingly, the overexpression of ABCB1 and ABCA2 is connected with various neurodegenerative pathologies, especially Alzheimer’s disease [51,52]. Therefore, the inhibition of acetylcholinesterase activity, which is also related to Alzheimer’s disease, may be a potential therapeutic target [53]. In our study, all the tested compounds proved their strong ability to inhibit the activity of acetylcholinesterase in a dose-dependent manner.

We have clearly demonstrated that silybin and dehydrosilybin act mostly at the receptor level, e.g., interacting with three-dimensional (3D) structures and not as mere antioxidants acting in isotropic milieu. The distinct activity of pure stereomers corroborates the general narrative that flavonolignans from silymarin are relatively poor antioxidants but they are strong effectors acting in terms of “para-hormesis” [31]. Unfortunately, current literature is “polluted” by the studies declaring silymarin as a “single compound” or entirely ignoring the fact that each flavonolignan from silymarin (except silydianin) exists in the form of two stereomers having totally different biological activities [54]. Our study is a clear proof-of-concept that exclusively, optically pure flavonolignan derivatives can be investigated in terms of biological activities.

## 5. Conclusions

The MDR phenomenon is a highly topical issue not only in cancer therapy but also in the treatment of other diseases. Therefore, regulation of P-glycoprotein and other ABC transporters involved is more than desirable. The use of secondary metabolites such as flavonolignans and flavonoids with high antioxidant, anti-inflammatory and anti-cancer activities accompanied with low negative side effects and negligible toxicity might be quite beneficial. Silybin and dehydrosilybin showed strong biological effects in several assays used. The antioxidant capacity of derivatives was confirmed by a classical biochemical assay (ORAC) and verified by a cellular assay in vitro. The cellular assay is more representative, also including parameters like bioavailability, metabolism, stability and others. In a dose-dependent manner, they modulated the production of inflammatory molecules (NO, TNF-α, IL-6). All the compounds inhibited acetylcholinesterase and P-gp efflux pump, which was also confirmed by sensitization of a resistant ovarian cell line. The direct inhibition of P-gp ATPase activity and also downregulation of the mRNA level of *ABC* gene expression was only observed by silybin B. Last but not least, the individual stereomers displayed different activities in most of the assays used, therefore confirming the importance of using optically pure stereomers in evaluation of biological activity of compounds with stereogenic centers.

## Figures and Tables

**Table 1:** Relative oxygen radical absorption capacity of silybin and dehydrosilybin diastereomers.

Compound	ORAC	CAA
Silybin A	6.6 ± 0.3 ^a^	9.8 ± 0.4 ^b,c^
Silybin B	6.8 ± 0.3 ^a^	8.9 ± 0.3 ^b^
Silybin AB	7.1 ± 0.6 ^a^	10.7 ± 0.9 ^c,d^
Dehydrosilybin A	11.4 ± 1.2 ^b^	11.8 ± 0.7 ^d^
Dehydrosilybin B	11.8 ± 0.6 ^b^	12.1 ± 0.4 ^d^
Dehydrosilybin AB	10.1 ± 0.0 ^b^	9.2 ± 0.5 ^b,c^
Quercetin	6.0 ± 0.7 ^a^	5.4 ± 0.3 ^a^

The data are presented as the concentration necessary to halve the fluorescence response (IC_50_, µM). ORAC: Oxygen radical absorption capacity; CAA: Cellular antioxidant activity assay. Data represent the average of concentration (IC_50_) of three repetitions with corresponding standard error of the mean. The data were analyzed with one-way analysis of variance (ANOVA, Statistica software (Tibco Software Inc., Tulsa, OK, USA)) and Duncan’s post hoc test, where the differences between groups were considered statistically significant when *p* < 0.05. Statistical significances are denoted by different letters.

**Table 2:** Anti-inflammatory activity of silybin and dehydrosilybin stereomers. Indomethacin represents the positive control.

Compound	NO Production
Silybin A	26.7 ± 1.6 ^a,b^
Silybin B	26.0 ± 3.0 ^a,b^
Silybin AB	23.6 ± 1.2 ^a,b^
Dehydrosilybin A	23.2 ± 1.4 ^a,b^
Dehydrosilybin B	31.3 ± 2.4 ^b^
Dehydrosilybin AB	19.1 ± 0.2 ^a^
Indomethacin	78.1 ± 7.5 ^c^

Data represent the average of concentrations necessary to halve NO production (IC_50_, µM) of three repetitions with corresponding standard errors of the mean. The data were analyzed with one-way analysis of variance (ANOVA, Statistica software (Tibco Software Inc., Tulsa, OK, USA)) and Duncan’s post hoc test, where the differences between groups were considered statistically significant when *p* < 0.05. Statistical significances are denoted by different letters.

**Table 3:** Inhibition of the release of pro-inflammatory cytokines TNF-α and IL-6 by silybin and dehydrosilybin stereomers.

Compound	TNF-α	IL-6
Silybin A	29.9 ± 1.35 ^c^	8.4 ± 0.3 ^d^
Silybin B	53.3 ± 0.7 ^f^	5.2 ± 0.2 ^b^
Silybin AB	38.7 ± 0.0 ^d^	4.8 ± 0.3 ^a,b^
Dehydrosilybin A	20.7 ± 0.4 ^a^	4.2 ± 0.2 ^a^
Dehydrosilybin B	44.2 ± 0.3 ^e^	7.0 ± 0.1 ^c^
Dehydrosilybin AB	26.7 ± 0.1 ^b^	15.4 ± 0.6 ^e^

Data represent the average of concentrations necessary to halve the release of TNF-α and IL-6 (IC_50_, µM) of three repetitions with corresponding standard errors of the mean. The data were analyzed with one-way analysis of variance (ANOVA, Statistica software (Tibco Software Inc., Tulsa, OK, USA)) and Duncan’s post hoc test, where the differences between groups were considered statistically significant when *p* < 0.05. Statistical significances are denoted by different letters.

**Table 4:** The effect of various concentrations of silybins and dehydrosilybins (12.5, 25, 50 µM) on the release of pro-inflammatory cytokine TNF-α by LPS-induced macrophages. Positive control represents the cells challenged with LPS and without pre-treatment of tested compounds.

TNF-α (pg/mL)	Concentration of Compound		
Compound	12.5 µM	25 µM	50 µM
Silybin A	2092 ± 48 ^a^	1476 ± 39 ^b^	415 ± 4 ^b^
Silybin B	2441 ± 68 ^b^	2375 ± 64 ^c^	1792 ± 63 ^e^
Silybin AB	2497 ± 37 ^b^	2425 ± 145 ^c^	1177 ± 75 ^d^
Dehydrosilybin A	2338 ± 63 ^ab^	777 ± 28 ^a^	0 ± 0 ^a^
Dehydrosilybin B	2528 ± 171 ^b^	2774 ± 84 ^d^	982 ± 25 ^c^
Dehydrosilybin AB	2617 ± 118 ^b^	2364 ± 108 ^c^	0 ± 0 ^a^
Non-treated LPS-induced cells (control)	2954 ± 92 ^c^	2954 ± 92 ^d^	2954 ± 92 ^e^

Data represent the average of three repetitions with corresponding standard errors of the mean. The data were analyzed with one-way analysis of variance (ANOVA, Statistica software (Tibco Software Inc., Tulsa, OK, USA)) and Duncan’s post hoc test, where the differences between groups were considered statistically significant when *p* < 0.05. Statistical significances are denoted by different letters.

**Table 5:** The effect of various concentrations of silybins and dehydrosilybins (12.5, 25, 50 µM) on the release of pro-inflammatory cytokine IL-6 by LPS-induced macrophages. Positive control represents the cells challenged with LPS without the presence of tested compounds.

IL-6 (pg/mL)	Concentration of Compound		
**Compound**	7 µM	15 µM	50 µM
Silybin A	422 ± 42 ^b^	271 ± 15 ^c^	125 ± 16 ^c^
Silybin B	297 ± 23 ^a^	166 ± 6 ^b^	37 ± 2 ^a^
Silybin AB	311 ± 18 ^a^	173 ± 5 ^b^	83 ± 4 ^b^
Dehydrosilybin A	292 ± 15 ^a^	79 ± 8 ^a^	0 ± 0 ^a^
Dehydrosilybin B	582 ± 19 ^c^	301 ± 9 ^c^	122 ± 3 ^b,c^
Dehydrosilybin AB	997 ± 41 ^d^	441 ± 19 ^d^	0 ± 0 ^a^
Non-treated LPS-induced cells (control)	1148 ± 35 ^e^	1148 ± 35 ^e^	1148 ± 35 ^d^

Data represent the average of three repetitions with corresponding standard errors of the mean. The data were analyzed with one-way analysis of variance (ANOVA, Statistica software (Tibco Software Inc., Tulsa, OK, USA)) and Duncan’s post hoc test, where the differences between groups were considered statistically significant when *p* < 0.05. Statistical significances are denoted by different letters.

**Table 6:** The effect of silybin and dehydrosilybin stereomers on the acetylcholinesterase activity and efflux pump activity.

Compound/Activity	P-gp	AChE
Silybin A	43.1 ± 6.4 ^c,d^	181.0 ± 6.1 ^e^
Silybin B	51.9 ± 2.1 ^d,e^	83.7 ± 6.4 ^d^
Silybin AB	32.6 ± 3.6 ^b,c^	22.6 ± 1.4 ^c^
Dehydrosilybin A	60.7 ± 10.0 ^e^	6.9 ± 0.6 ^b^
Dehydrosilybin B	40.7 ± 3.8 ^c,d^	6.8 ± 0.8 ^b^
Dehydrosilybin AB	21.1 ± 0.6 ^a,b^	2.7 ± 0.3 ^b^
Orthovanadate	18.9 ± 1.8 ^a^	-
Eserin	-	0.01 ± 0.0 ^a^

AChE: acetylcholinesterase, Eserin: reversible inhibitor of acetylcholinesterase; P-gp: transmembrane efflux pump. The P-gp assay was realized using the commercial P-gp–Glo assay system (Promega, Madison, WI, USA), including a membrane fraction with recombinant human P-gp. The AChE assay was realized as a classical enzymatic assay. Data represent the average concentration necessary to halve ATP consumption/acetylcholinesterase activity (IC_50_, µM) of three values of repetitions, with corresponding standard error of the mean. The data were analyzed with one-way analysis of variance (ANOVA, Statistica software (Tibco Software Inc., Tulsa, OK, USA)) and Duncan’s post hoc test, where the differences between groups were considered statistically significant when *p* < 0.05. Statistical significances are denoted by different letters.

**Table 7:** The impact of silybin and dehydrosilybin stereomers on the viability of doxorubicin-resistant human ovarian adenocarcinoma cell line.

Compound	IC_50_ (µM)
Silybin A	251.5 ± 7.6 ^e^
Silybin B	194.3 ± 8.8 ^d^
Silybin AB	197.4 ± 4.0 ^d^
Dehydrosilybin A	74.4± 4.8 ^c^
Dehydrosilybin B	27.1 ± 0.4 ^b^
Dehydrosilybin AB	33.6 ± 0.4 ^b^
Doxorubicin	1.88 ± 0.07 ^a^

Data represent the average concentration necessary to halve cell viability (IC_50_, µM) of three repetitions with corresponding standard error of the mean. The data were analyzed with one-way analysis of variance (ANOVA, Statistica software (Tibco Software Inc., Tulsa, OK, USA)) and Duncan’s post hoc test, where the differences between groups were considered statistically significant when *p* < 0.05. Statistical significances are denoted by different letters.

**Table 8:** The sensitization effect of silybin and dehydrosilybin stereomers on the effective dose of doxorubicin inhibiting the viability of doxorubicin-resistant human ovarian carcinoma cells (A2780/DOX).

Compound	10 µM	20 µM	30 µM	50 µM
Silybin A	1.40 ± 0.10 ^b^	1.17 ± 0.08 ^b^	0.99 ± 0.04 ^b^	0.52 ± 0.02 ^b^
Silybin B	1.37 ± 0.00 ^b^	1.22 ± 0.10 ^b^	1.04 ± 0.01 ^b^	0.56 ± 0.05 ^b^
Silybin AB	1.35 ± 0.02 ^b^	1.20 ± 0.02 ^b^	1.11 ± 0.05 ^b^	0.24 ± 0.00 ^a^
Dehydrosilybin A	0.91 ± 0.08 ^a^	0.75 ± 0.02 ^a^	0.20 ± 0.01 ^a^	-
Dehydrosilybin B	0.95 ± 0.02 ^a^	-	-	-
Dehydrosilybin AB	0.77 ± 0.03 ^a^	-	-	-
Doxorubicin	1.88 ± 0.07 ^c^			

Data represent the average concentration (IC_50_) of three repetitions with corresponding standard error of the mean. The data were analyzed with one-way analysis of variance (ANOVA, Statistica software (Tibco Software Inc., Tulsa, OK, USA)) and Duncan’s post hoc test, where the differences between groups were considered statistically significant when *p* < 0.05. Statistical significances are denoted by different letters.

**Table 9:** Doxorubicin-sensitization rate of silybin and dehydrosilybin isomers in A2780/DOX cell line.

Compound	10 µM	20 µM	30 µM	50 µM
Silybin A	(1.3 ± 0.1) ×	(1.6 ± 0.2) ×	(1.9 ± 0.1) ×	(3.6 ± 0.3) ×
Silybin B	(1.4 ± 0.1) ×	(1.5 ± 0.2) ×	(1.8 ± 0.1) ×	(3.3 ± 0.4) ×
Silybin AB	(1.4 ± 0.1) ×	(1.6 ± 0.1) ×	(1.7 ± 0.1) ×	(7.7 ± 0.4) ×
Dehydrosilybin A	(2.1 ± 0.3) ×	(2.5 ± 0.2) ×	(9.5 ± 0.7) ×	-
Dehydrosilybin B	(2.0 ± 0.1) ×	-	-	-
Dehydrosilybin AB	(2.4 ± 0.2) ×	-	-	-

The sensitization rate was determined as the ratio of IC_50_ values of doxorubicin and doxorubicin + tested compounds.

## Abbreviations

A549	human lung cancer cell line
A2780/DR	human ovarian carcinoma resistant to doxorubicin
A2780/DDP	human ovarian carcinoma resistant to cisplatin and taxol
AAPH	2,2′-azo-*bis*(2-methylpropionamidine) dihydrochloride
ABC	ATP binding cassette
AChE	Acetylcholinesterase
ATC	Acetylcholine chloride
BACH1	Transcription regulator protein
Bcl-2	Apoptosis regulation family of proteins
BRCP	Breast cancer resistance protein
CAA	cellular antioxidant activity
COX-2	cyklooxynase-2
Ct	Cycle threshold
DCFH-DA	2′,7′-dichlorofluorescin diacetate
DOX	doxorubicin
DPPH	2,2-diphenyl-1-picrylhydrazyl
DMEM	Dulbecco’s Modified Eagle’s Medium—high glucose
DTNB	5,5′-dithio-*bis*-2-nitrobenzoic acid
EGFR	Epidermal Growth Factor Receptor
EMEM	Eagle´s Minimum Essential Medium
FBS	fetal bovine serum
HaCaT	human keratinocyte cell line
HepG2	human hepatocellular adenocarcinoma
HSC	hepatic stellate cells
IL-1β	Interleukin-1β
IL-2	Interleukin-2
IL-6	Interleukin-6
INF-ƴ	Interferon-ƴ
Kras	Proto-Oncogene, GTPase
LP	lipid peroxidation
LPS	lipopolysaccharides
MCF-7	Breast cancer cell line
MEM	Essential Medium Eagle, no phenol red
MDR	Multi-drug resistance
MRP1	Multi-drug resistance-associated protein 1
MRP2	Multi-drug resistance-associated protein 21
mtDNA	mitochondrial DNA
NF-ĸB	nuclear factor κ-light-chain-enhancer of activated B cells
Nrf2	nuclear factor erythroid 2-related factor 2
NHDF	primary human dermal fibroblasts
ORAC	oxygen radical absorption capacity
PBMCs	peripheral blood mononuclear cells
PBS	phosphate-buffered saline
PDGF	Platelet-Derived Growth Factor
P-g	P-glycoprotein
RAW 264.7	mouse macrophages
RNS	reactive nitrogen species
ROS	reactive oxygen species
RLU	relative luminescence units
SKOV-3	human adenocarcinoma cell line
TGF-β	transforming growth factor β
TNF-α	tumor necrosis factor α
VPA17	small lung carcinoma resistant to etoposide, doxorubicin and vincristine

## Appendix A

**Table A1:** Effect of silybin and dehydrosilybin isomers on expression of ABC (ATP-binding cassette) superfamily genes in doxorubicin-resistant ovarian carcinoma (A2780/DOX).

	Silybin A			Silybin B			Silybin AB			Dehydrosilybin A			Dehydrosilybin B			Dehydrosilybin AB		
Gene Symbol	Expression Difference	*p*	Treated vs. Untreated	Expression Difference	*p*	Treated vs. Untreated	Expression Difference	*p*	Treated vs. Untreated	Expression Difference	*p*	Treated vs. Untreated	Expression Difference	*p*	Treated vs. Untreated	Expression Difference	*p*	Treated vs. Untreated
PPI																		
ABCA1							2.021	0.000	UP							1.803	0.000	UP
ABCA2	1.078	0.000	UP	0.520	0.000	DOWN	2.583	0.000	UP	0.778	0.000	DOWN	0.891	0.000	DOWN			
ABCA3				0.228	0.000	DOWN												
ABCA5				0.356	0.000	DOWN	1.933	0.000	UP									
ABCA7							2.704	0.000	UP									
ABCB1				0.208	0.000	DOWN												
ABCB2	1.141	0.000	UP							1.905	0.000	UP	1.625	0.000	UP	2.341	0.000	UP
ABCB3				0.365	0.000	DOWN												
ABCB6				0.396	0.038	DOWN	1.838	0.000	UP									
ABCB7							1.803	0.000	UP							1.463	0.000	UP
ABCB8				0.413	0.000	DOWN	1.503	0.000	UP									
ABCB9				0.392	0.000	DOWN	2.091	0.000	UP	1.317	0.000	UP				1.669	0.000	UP
ABCB10	1.136	0.027	UP	0.329	0.000	DOWN	2.204	0.035	UP	1.236	0.028	UP	1.186	0.030	UP	1.589	0.038	UP
ABCC1				0.413	0.000	DOWN	2.521	0.000	UP									
ABCC2				0.341	0.038	DOWN												
ABCC5				0.365	0.000	DOWN	1.819	0.000	UP							1.519	0.000	UP
ABCC10	1.260	0.027	UP	0.340	0.000	DOWN	1.857	0.035	UP	1.200	0.028	UP	1.107	0.030	UP	1.471	0.038	UP
ABCD1	1.187	0.048	UP	0.348	0.000	DOWN				1.063	0.120							
ABCD2	1.063	0.027	UP	0.322	0.000	DOWN	1.566	0.035	UP	1.205	0.028	UP	0.860	0.000	DOWN	1.199	0.038	UP
ABCD3				0.337	0.038	DOWN	2.181	0.000	UP							1.545	0.000	UP
ABCD4				0.370	0.000	DOWN	1.882	0.035	UP									
ABCE1				0.391	0.000	DOWN	1.558	0.000	UP							1.408	0.000	UP
ABCF1				0.260	0.000	DOWN										0.322	0.000	DOWN
ABCF2				0.307	0.000	DOWN	1.326	0.035	UP	1.198	0.028	UP	1.334	0.030	UP	1.418	0.038	UP
ABCF3				0.328	0.000	DOWN	1.440	0.000	UP	1.602	0.000	UP				1.706	0.000	UP
ABCG1				0.303	0.000	DOWN	2.416	0.000	UP									
ABCG2				0.335	0.038	DOWN										1.921	0.000	UP
ABCG4	1.185	0.000	UP	0.366	0.000	DOWN	1.846	0.000	UP	0.950	0.000	DOWN	0.736	0.000	DOWN	1.162	0.000	UP

**Table A2:** Effect of silybin and dehydrosilybin isomers combined with doxorubicin at IC_25_ on expression of ABC (ATP-binding cassette) superfamily genes in doxorubicin-resistant ovarian carcinoma (A2780/DOX).

	Silybin A			Silybin B			Silybin AB			Dehydrosilybin A			Dehydrosilybin B			Dehydrosilybin AB		
Gene Symbol	Expression Difference	*p*	Treated vs. Untreated	Expression Difference	*p*	Treated vs. Untreated	Expression Difference	*p*	Treated vs. Untreated	Expression Difference	*p*	Treated vs. Untreated	Expression Difference	*p*	Treated vs. Untreated	Expression Difference	*p*	Treated vs. Untreated
PPI	1.000			1.000				1.000			1.000					1.000		
ABCA1	2.702	0.000	UP	2.415	0.000	UP	2.922	0.000	UP	4.172	0.000	UP	4.412	0.000	UP	3.122	0.000	UP
ABCA2	1.239	0.000	UP	0.903	0.000	DOWN	1.207	0.000	UP	1.105	0.000	UP	1.078	0.000	UP	1.046	0.000	UP
ABCA3				0.794	0.000	DOWN	1.142	0.064		1.042	0.455		1.060	0.116		0.943	0.061	
ABCA5	2.427	0.000	UP	1.821	0.000	UP	2.122	0.000	UP	1.223	0.000	UP	1.707	0.000	UP	1.254	0.000	UP
ABCB2	17.975	0.000	UP	15.698	0.000	UP	18.114	0.000	UP	5.557	0.000	UP	9.286	0.000	UP	5.145	0.000	UP
ABCB6	1.665	0.000	UP				1.527	0.000	UP	1.149	0.059		1.343	0.059		1.262	0.061	
ABCB9	1.586	0.000	UP	1.241	0.000	UP	1.638	0.000	UP	1.109	0.059		1.218	0.000	UP	1.149	0.061	
ABCB10	1.060	0.032	UP	0.835	0.000	DOWN	1.069	0.039	UP	1.020	0.040	UP	1.221	0.035	UP	1.119	0.025	UP
ABCC1	1.711	0.000	UP				1.640	0.000	UP	0.907	0.388		1.213	0.059		1.301	0.061	
ABCC5	1.782	0.000	UP	1.473	0.000	UP	1.787	0.000	UP	1.116	0.397		1.385	0.059		1.288	0.061	
ABCC10	1.812	0.032	UP	1.349	0.036	UP	1.855	0.039	UP	1.214	0.040	UP	1.618	0.035	UP	1.323	0.025	UP
ABCD1							2.868	0.064		1.435	0.067		1.784	0.056		1.412	0.042	UP
ABCD2	0.426	0.000	DOWN	0.448	0.000	DOWN	0.494	0.000	DOWN	0.698	0.000	DOWN	0.733	0.000	DOWN	0.712	0.000	DOWN
ABCD3	1.637	0.000	UP				1.560	0.000	UP	1.043	0.397		1.347	0.000	UP	1.288	0.000	UP
ABCF1							0.137	0.000	DOWN	0.920	0.397		0.784	0.059		0.923	0.400	
ABCF2	1.242	0.032	UP				1.463	0.039	UP	1.044	0.113		1.226	0.035	UP	1.343	0.025	UP
ABCF3	1.570	0.000	UP	1.335	0.000	UP	1.554	0.000	UP	1.096	0.059		1.963	0.000	UP	1.326	0.000	UP
ABCG1							1.559	0.073		2.714	0.000	UP	2.931	0.000	UP	1.826	0.056	
ABCG2	2.415	0.000	UP	1.902	0.000	UP	2.373	0.000	UP	1.259	0.073		1.948	0.000	UP	1.698	0.000	UP
ABCG4	4.751	0.000	UP	4.110	0.000	UP	5.289	0.000	UP	1.992	0.000	UP	2.860	0.000	UP	2.008	0.000	UP
